# Causal relationship between gut microbiota and rosacea: a two-sample Mendelian randomization study

**DOI:** 10.3389/fmed.2024.1322685

**Published:** 2024-03-22

**Authors:** Jiaqi Li, Fengjuan Yang, Yuling Liu, Xian Jiang

**Affiliations:** ^1^Department of Dermatology, West China Hospital, Sichuan University, Chengdu, China; ^2^Laboratory of Dermatology, Clinical Institute of Inflammation and Immunology, Frontiers Science Center for Disease-related Molecular Network, West China Hospital, Sichuan University, Chengdu, China; ^3^Med-X Center for Informatics, Sichuan University, Chengdu, China

**Keywords:** Mendelian randomization, gut microbiota, rosacea, therapy, dermatology

## Abstract

**Background:**

Rosacea, a chronic inflammatory skin condition affecting millions worldwide, is influenced by complex interactions between genetic and environmental factors. Although gut microbiota’s role in skin health is well-acknowledged, definitive causal links between gut microbiota and rosacea remain under-explored.

**Methods:**

Using a two-sample Mendelian randomization (MR) design, this study examined potential causal relationships between gut microbiota and rosacea. Data was sourced from the largest Genome-Wide Association Study (GWAS) for gut microbiota and the FinnGen biobank for rosacea. A total of 2078 single nucleotide polymorphisms (SNPs) associated with gut microbiota were identified and analyzed using a suite of MR techniques to discern causal effects.

**Results:**

The study identified a protective role against rosacea for two bacterial genera: phylum Actinobacteria and genus Butyrivibrio. Furthermore, 14 gut microbiota taxa were discovered to exert significant causal effects on variant categories of rosacea. While none of these results met the strict False Discovery Rate correction threshold, they retained nominal significance. MR outcomes showed no pleiotropy, with homogeneity observed across selected SNPs. Directionality tests pointed toward a robust causative path from gut microbiota to rosacea.

**Conclusion:**

This study provides compelling evidence of the gut microbiota’s nominal causal influence on rosacea, shedding light on the gut-skin axis’s intricacies and offering potential avenues for therapeutic interventions in rosacea management. Further research is warranted to validate these findings and explore their clinical implications.

## Introduction

Rosacea, marked by persistent facial redness, flushing, papules, and phymatous alterations, is a chronic inflammatory skin condition affecting nearly 40 million individuals globally (1, 2). This condition profoundly affects the quality of life, emotional well-being, and self-perception of those who suffer from it. The root cause of rosacea remains multifaceted and elusive. It likely stems from a blend of genetic factors and environmental influences, causing irregularities in both the innate and adaptive immune responses. Moreover, it is believed to cause dysfunction in the neurovascular reactions seen in rosacea patients (3).

In recent years, the role of microbiota in regulating immunity has emerged as a key focal point in research regarding the development and progression of rosacea. Multiple studies comparing the skin microbiota composition of rosacea patients with that of the general population have yielded varied results (4, 5). The gut microbiota, a diverse group of microorganisms in the gastrointestinal tract, plays a pivotal role in human health. It regulates metabolism, shields against pathogens, and shapes the immune system. The concept of the gut-skin axis is gaining attention. This suggests the gut microbiota’s role in maintaining skin balance is integral. Conditions like acne, atopic dermatitis, and psoriasis have all been linked to disruptions in gut microbiota (6–8). Notably, research in Asian cohorts has revealed variations in gut microbiota between those with rosacea and their healthy counterparts, pointing toward a potential relationship (9). In one study, patients with a papulopustular rash, including a significant number with rosacea, showed better improvement when treated with the *Escherichia coli* Nissen 1917 oral probiotic alongside regular topical treatments than with the standard treatment alone (10). However, a vital question remains: Is this relationship simply observational, or does gut microbiota imbalance directly influence the emergence or worsening of rosacea? The interplay between the two is intricate, with factors like age, gender, and body mass index affecting gut microbiota, making it challenging to gather a large enough sample for meaningful analysis. Furthermore, ethical considerations limit clinical trials aiming to establish a definitive link between gut microbiota and rosacea.

The Mendelian randomization (MR) methodology harnesses genetic variants as instrumental variables to unravel causal associations between modifiable exposures or risk factors and clinical outcomes (11). Given that the prerequisites for instrumental variables are satisfied, the derived estimator remains robust, sidestepping challenges related to unobserved confounding and reverse causation (12). MR presents several advantages over randomized controlled trials (RCTs), particularly concerning feasibility and the mitigation of confounding bias inherent in observational research. By utilizing genetic variants that are randomly assigned at conception and associated with the exposure, it helps to mitigate confounding factors and reverse causation, thus providing more robust evidence of a causal relationship. Yet, the potential causal association between gut microbiota and rosacea has not been explored using the MR. In response to this gap, our investigation adopted a two-sample MR approach to elucidate this prospective causal link.

## Materials and methods

### Study design

**Figure 1:**
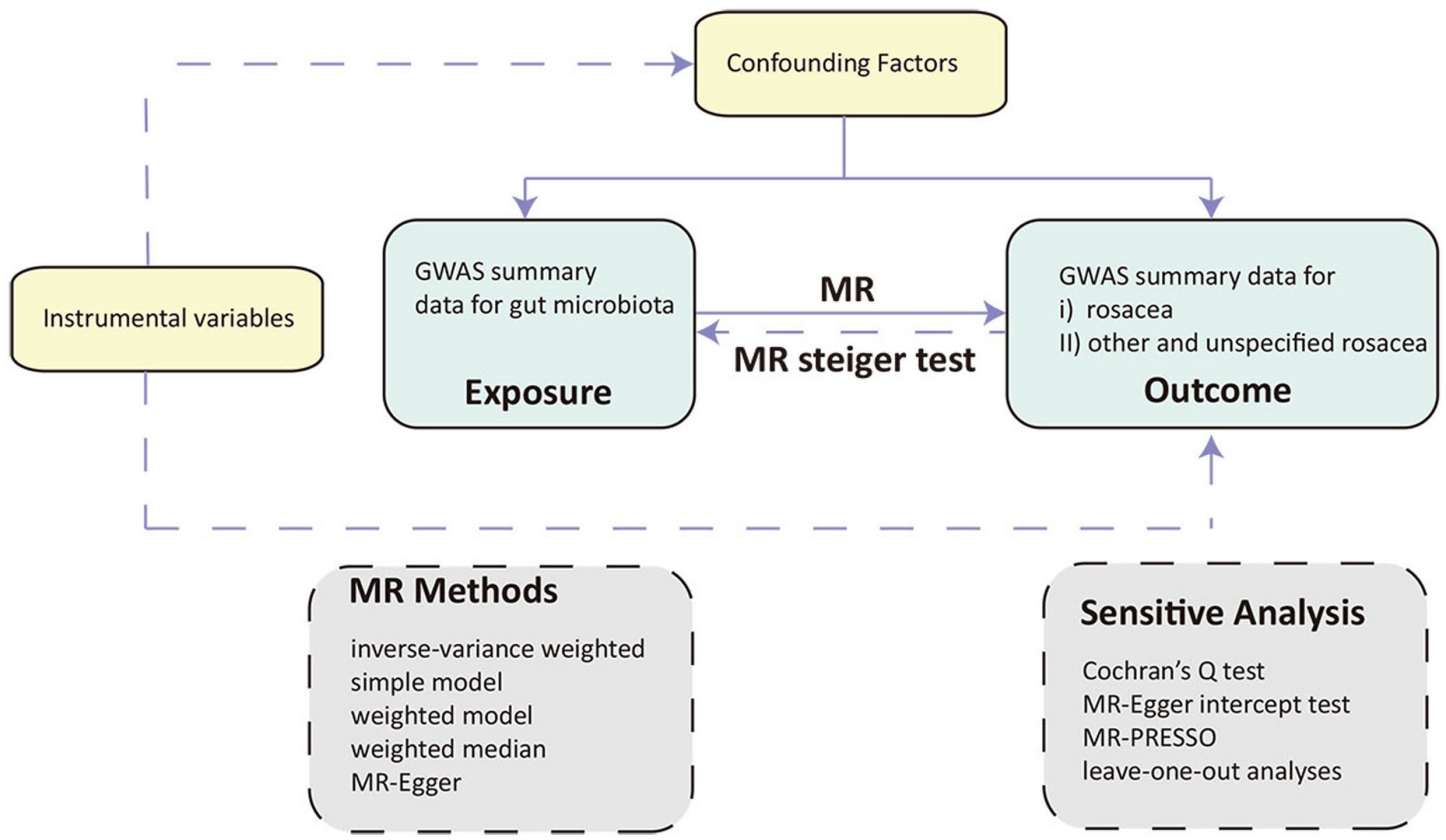
Overview of the Mendelian randomization (MR) framework involves three core principles. First, the instrumental variables (IVs) must have a strong association with the exposure of interest. Second, these IVs should not be associated with any confounding factors that might influence the observed relationship between exposure and outcome. Lastly, the relationship between IVs and the outcome should be mediated exclusively through the exposure, without any direct links. MR, Mendelian randomization.

We performed a two-sample MR design to uncover the possible causal effects between gut microbiota and rosacea, In adherence to the principles of MR, we established three fundamental hypotheses for our trial, each pivotal in assessing causality and unveiling the relationship between gut microbiota and rosacea: (i) Relevance hypothesis: Meeting this criterion ensures that the genetic instruments utilized in our MR analysis effectively represent the intended exposure, allowing us to gage the impact of gut microbiota on rosacea development. (ii) Independence hypothesis: By confirming the independence of our genetic instruments from these confounding factors, we can minimize the risk of spurious associations and enhance the validity of our causal inferences. (iii) Exclusion restriction hypothesis: Validating the exclusion restriction assumption is essential in establishing a direct and unambiguous link between gut microbiota alterations and rosacea, reinforcing the causal interpretation of our MR analysis (Figure 1) (13).

### Data source

Genetic markers linked to the composition of the gut microbiota were sourced from the most comprehensive genome-wide meta-analysis to date on gut microbiota composition by the MiBioGen consortium. This analysis encompassed 18,340 participants across 24 cohorts, predominantly of European descent (14). This extensive dataset included 211 bacterial taxa units, encompassing 131 genera, 35 families, 20 orders, 16 classes, and 9 phyla. The richness and comprehensiveness of this dataset provided a robust basis for our MR study. After excluding 15 unidentified taxa, 196 were retained for subsequent MR analyses. Simultaneously, the data relevant to rosacea cases was sourced from the FinnGen biobank and categorized into two distinct datasets. The first, termed ‘finn-b-L12_ROSACEA,’ emerged from the FinnGen biobank’s 7th analysis round, and the cases were defined by L71 in ICD 10. This dataset comprises 1,195 documented rosacea cases, juxtaposed with a control cohort of 211,139 individuals. The second dataset, tagged as ‘finn-b-L12_ROSACEANAS,’ was extracted from the FinnGen biobank’s 9th analysis round. It is defined by L71.8 and L71.9 in ICD 10 and 6953A in ICD 9, which incorporates 2,210 cases, each identified as either ‘other’ or ‘unspecified’ rosacea types, paired with a comprehensive control set of 361,140 individuals. The utilization of these diverse and comprehensive sources of data will undoubtedly facilitate a meticulous and thorough investigation into delineating the potential causal relationship that exists between gut microbiota and rosacea.

### Instrumental variable selection

To identify potential instrumental variables (IVs), we initiated the selection process by considering SNPs associated with gut bacterial taxa at the genome-wide significance threshold of *p* < 1.0 × 10^−5^. The chosen IVs needed to satisfy specific quality control criteria: First, we set the LD threshold for clumping at r^2 < 0.001 and a window size of 10,000 kb to minimize the influence of LD on the results. This step aimed to ensure that the selected SNPs were relatively independent and not in strong LD with each other. Next, we harmonized the effect estimates for both exposure and outcome variants, excluding any potential SNPs with incompatible alleles or palindromic characteristics. In pursuit of consistency, only SNPs available for all examined traits were utilized as IVs, with no proxies used to substitute those absent in outcome data. Third, each of the selected SNPs was meticulously reviewed using PhenoScanner V2.^1^ This tool provides detailed information on SNP phenotypes, helping to determine whether the SNPs affect the outcomes solely through their exposure. Lastly, to gage the robustness of our selected instruments, we computed the F statistic using the formula: F = (β/SE)^2, where β represents the effect size and SE stands for the standard error of the effect size, and criterion of *F* > 10 was upheld, aligning with the principle of not exhibiting bias toward weak IVs (15).

### MR analysis

For our foundational analysis, we employed a suite of MR techniques to discern causal effects. This ensemble included the inverse-variance weighted (IVW) method, which served as our cornerstone, supplemented by the simple model, weighted model, weighted median, and MR-Egger methods. Together, these techniques facilitated a rigorous appraisal of causal connections.

To address the potential bias introduced by pleiotropy, we turned to the intercept term of the MR-Egger regression. An intercept term nearing zero indicates the absence of horizontal pleiotropy in the particular SNP under investigation in our bidirectional MR approach (16). To delve deeper into this phenomenon, we harnessed the MR-PRESSO global test, aiming to discern any horizontal pleiotropy where a singular genetic variant might influence an array of traits, muddling the causative assessments (17). Heterogeneity within the IVW method was scrutinized using Cochran’s Q statistics, enriched by a nuanced assessment of funnel plots. Such tools shed light on the consistency and dependability of our findings. Moreover, a “leave-one-out” sensitivity analysis was performed to gage the bearing of singular SNPs on the foundational causal relationship, aiding in pinpointing potential biases tied to distinct genetic markers. With the expansive hypothesis testing at play, we scrupulously implemented the Benjamini-Hochberg (BH) procedure to adjust for concurrent comparisons in our forward analysis. A result boasting a False Discovery Rate *p* value (PFDR) below 0.1 was tagged as significant, marking a rigorous benchmark for significance. Concurrently, outcomes with a p value less than 0.05, but with a PFDR surpassing 0.1, were deemed to be of nominal significance. Such outcomes, while not reaching our stringent significance yardstick, underscored emerging trends worthy of deeper exploration. Finally, the MR Steiger test was initiated to robustly probe the causality direction between the exposure and the outcome, providing indispensable clarity on the causative trajectory.

## Results

**Figure 2:**
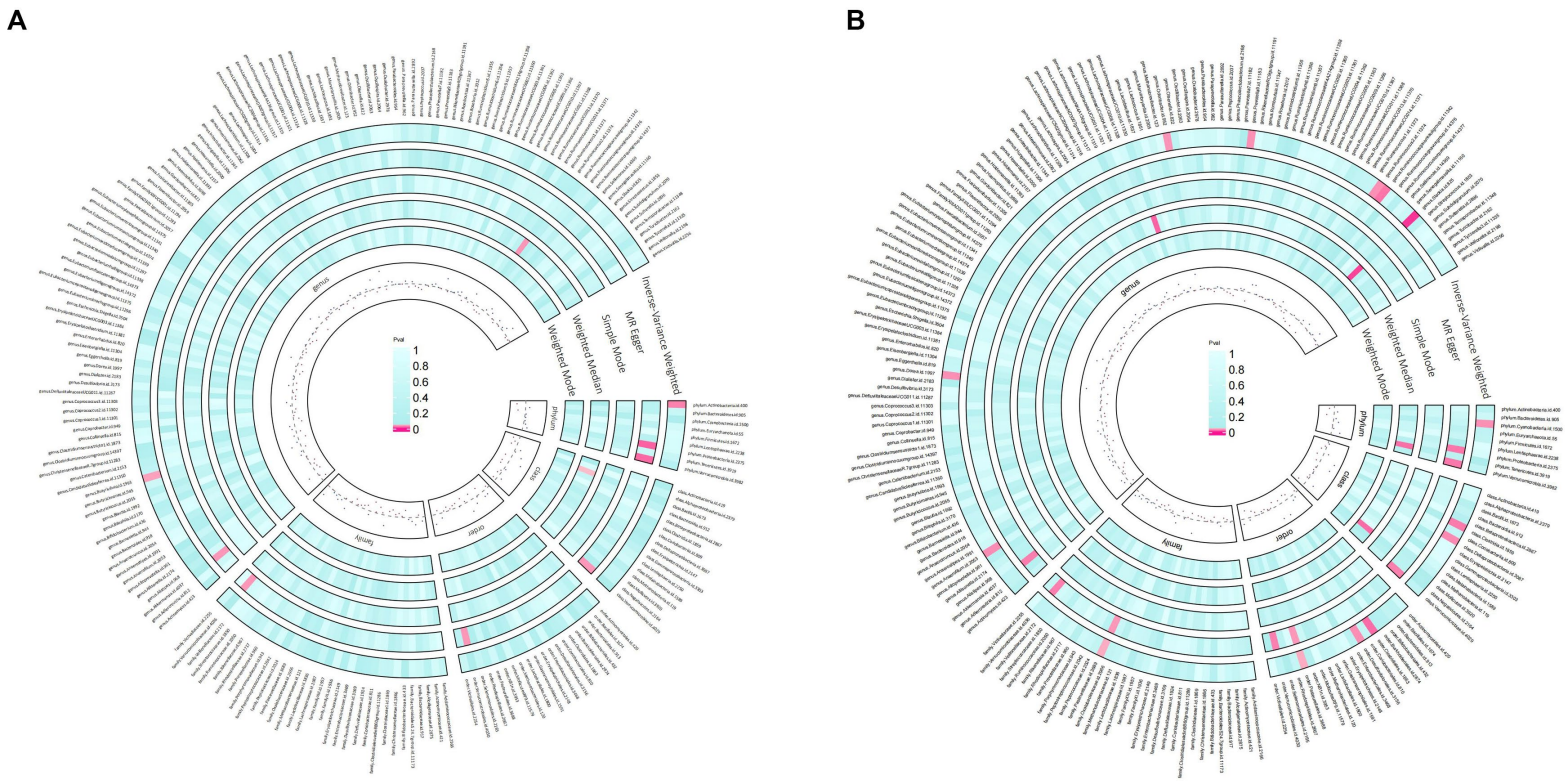
Preliminary MR estimates for the associations between gut microbiota and rosacea **(A)** and other or unspecified rosacea **(B)**.

**Figure 3:**
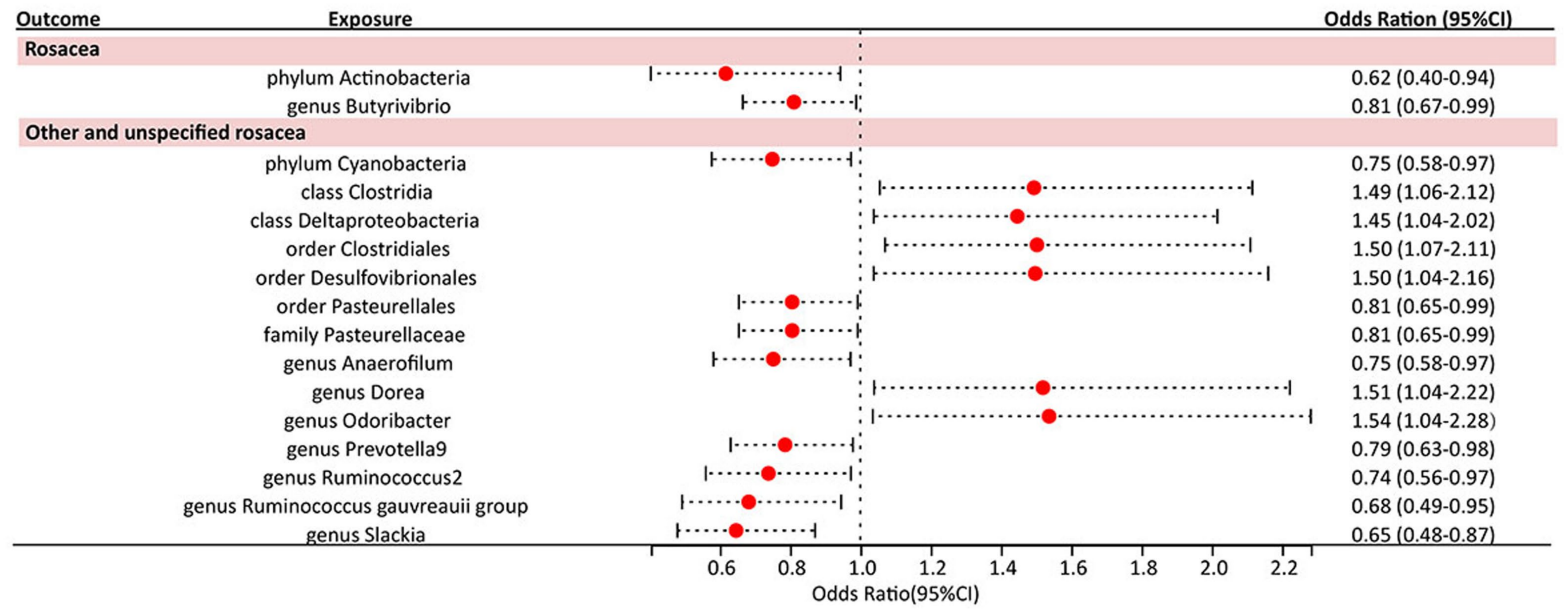
Forest plot of the causal association between gut microbiota and rosacea.

Under the screening threshold of *p* < 1 × 10^−5, we identified 2078 SNPs associated with gut microbiota. We illustrate our exploration of the association between gut microbiota and rosacea using five MR methods (Figure 2; Supplementary Tables S1, S2). All the F-statistics of IVs were larger than 10, which indicated weak instrument bias was unlikely. The IVW methods highlighted two bacterial genera: phylum *Actinobacteria* (OR=0.62, 95%CI: 0.40–0.94, *p*=0.026) and genus *Butyrivibrio* (OR=0.81, 95%CI: 0.67–0.99, *p*=0.038; Table 1). These demonstrated a protective role by restraining the onset of rosacea. Additionally, our examination uncovered 14 gut microbiota taxa exerting significant causal effects on other and unspecified rosacea, and range of taxa from broad phyla to specific genera was identified, with associations as detailed in Figure 3. The above results are depicted by scatter plots in Supplementary Figure S1 and illustrated through forest plots for causal effects of gut microbiota on rosacea risk with individual SNPs in Supplementary Figure S2. Despite the observed associations, no MR outcomes met the False Discovery Rate (FDR) correction threshold for multiple testing (Q_FDR_ < 0.1). Nevertheless, with *p*-values <0.05, these results hold nominal significance table.

**Table 1:** Significant MR analysis results of causal links between gut microbiome and rosacea using IVW method.

Exposure	Outcome	No.SNP	*β*	SE	P value	P_FDR_ value
Phylum *Actinobacteria*	Rosacea	14	−0.483	0.216	0.026	0.864
Genus *Butyrivibrio*		15	−0.210	0.101	0.038	0.864
Phylum *Cyanobacteria*	Other and unspecified rosacea	8	−0.288	0.133	0.031	0.960
Class *Clostridia*		11	0.401	0.177	0.024	0.951
Class *Deltaproteobacteria*		12	0.369	0.169	0.029	0.958
Order *Clostridiales*		12	0.407	0.173	0.019	0.943
Order *Desulfovibrionales*		11	0.403	0.187	0.031	0.960
Order *Pasteurellales*		13	−0.216	0.106	0.042	0.970
Family *Pasteurellaceae*		13	−0.216	0.106	0.042	0.970
Genus *Anaerofilum*		10	−0.285	0.131	0.030	0.959
Genus *Dorea*		10	0.418	0.194	0.031	0.960
Genus *Odoribacter*		7	0.430	0.202	0.033	0.962
Genus *Prevotella9*		15	−0.241	0.112	0.031	0.962
Genus *Ruminococcus2*		15	−0.303	0.141	0.031	0.960
Genus *Ruminococcus gauvreauii group*		11	−0.382	0.166	0.022	0.949
Genus *Slackia*		6	−0.436	0.152	0.004	0.820

Delving into the homogeneity of the data, Cochrane’s Q test affirmed the absence of noteworthy heterogeneity across the selected SNPs (*p* > 0.05; Table 2). Pleiotropy tests, including the MR Egger, signified no presence of pleiotropy in our study outcomes (all *p* > 0.05; Table 3). Supplementary investigations, like the leave-one-out analysis, hinted that certain individual SNPs might introduce biases in genetic predictions, as visualized in Supplementary Figure S3. Concurrently, the MR-PRESSO analysis and corroborated the absence of horizontal pleiotropy in our MR outcomes (all *p* > 0.05; Table 3). Crucially, the MR Steiger directionality tests unanimously indicated a potent causative trajectory from the gut microbiota toward rosacea across all evaluated outcomes, as detailed in Supplementary Table S3.

**Table 2:** Heterogeneity results from the Cochran’s Q test of significant causal links between gut microbiome and rosacea.

Exposure	Outcome	IVW		MR-Egger	
		Q	*p* value	Q	*p* value
Phylum *Actinobacteria*	Rosacea	10.572	0.647	10.138	0.604
Genus *Butyrivibrio*		16.491	0.289	16.327	0.232
Phylum *Cyanobacteria*	Other and unspecified rosacea	4.799	0.684	4.780	0.572
Class *Clostridia*		8.793	0.552	7.072	0.630
Class *Deltaproteobacteria*		11.266	0.421	11.224	0.340
Order *Clostridiales*		10.037	0.527	9.458	0.489
Order *Desulfovibrionales*		11.623	0.311	11.520	0.242
Order *Pasteurellales*		10.845	0.542	8.077	0.706
Family *Pasteurellaceae*		10.845	0.542	8.077	0.706
Genus *Anaerofilum*		14.500	0.106	14.499	0.070
Genus *Dorea*		8.403	0.494	8.402	0.395
Genus *Odoribacter*		4.532	0.605	4.532	0.476
Genus *Prevotella9*		14.926	0.383	14.926	0.312
Genus *Ruminococcus2*		17.106	0.251	17.106	0.195
Genus *Ruminococcus gauvreauii group*		11.653	0.309	10.843	0.287
Genus *Slackia*		3.768	0.583	1.589	0.811

**Table 3:** Pleiotropy results from Egger intercept analysis and MR presso.

Exposure	Outcome	MR egger-intercept		MR Presso Global
		Intercept	*p* value	
Phylum *Actinobacteria*	Rosacea	0.036	0.522	0.693
Genus *Butyrivibrio*		0.016	0.802	0.316
Phylum *Cyanobacteria*	Other and unspecified rosacea	0.008	0.894	0.733
Class *Clostridia*		−0.066	0.222	0.555
Class *Deltaproteobacteria*		0.010	0.851	0.447
Order *Clostridiales*		−0.037	0.464	0.528
Order *Desulfovibrionales*		0.015	0.783	0.305
Order *Pasteurellales*		0.045	0.124	0.563
Family *Pasteurellaceae*		0.045	0.124	0.589
Genus *Anaerofilum*		−3.9E-04	0.996	0.153
Genus *Dorea*		0.001	0.971	0.55
Genus *Odoribacter*		−1.7E-04	0.997	0.652
Genus *Prevotella9*		−1.4E-04	0.997	0.436
Genus *Ruminococcus2*		−0.041	0.434	0.344
Genus *Ruminococcus gauvreauii group*		5.39E-05	0.999	0.291
Genus *Slackia*		−0.146	0.214	0.621

## Discussion

The complex interplay between the gut microbiota and various health outcomes has been a focal point of numerous investigations in recent years. Our study ventured into this ever-evolving frontier to delineate the causal relationships between the gut microbiota and rosacea. Whether these gut microbiota features are the cause of or a result of the progression of rosacea is a question that is explored for the first time in this paper. Our findings underscore a promising direction in understanding the etiopathogenesis of rosacea and open new vistas for potential therapeutic interventions.

For the GWAS data related to rosacea, we selected two different datasets from FinnGen. When considered as outcomes of gut microbiota exposure, the two datasets yielded markedly different results. However, it is regrettable that the official website did not provide detailed descriptions for ‘rosacea’ and ‘other and unspecified rosacea’. It is well-established that rosacea can manifest in four phenotypes which are erythematotelangiectatic rosacea, papulopustular rosacea, phymatous rosacea, and ocular rosacea (18, 19). Past research has identified significant variations in the facial microbiota characteristics among these rosacea phenotypes, and it is reported skin microbiota in erythematotelangiectatic rosacea showed a depletion of *Roseomonas mucosa*, and papulopustular rosacea exhibited an enrichment in *Campylobacter ureolyticus* and *Corynebacterium kroppenstedtii* (5). Hence, we hypothesize that similar distinctions between phenotypes might also be present within the gut microbiota, and this could potentially account for the differing MR analysis results observed for ‘rosacea’ and ‘other and unspecified rosacea’. In this study, we identified 16 gut microbiota taxa that demonstrated a nominal causal association with rosacea. Of these, eight taxa exhibited protective causal relationships.

The human gastrointestinal tract undergoes a significant microbial colonization process immediately post-birth (20). The gut microbiome engages with the host immune system in a complex manner. It not only fosters immune tolerance toward dietary and environmental antigens but also directly defends against external pathogens by competitively binding to endothelial cells and initiating immune protective responses (21, 22). The gut microbiota of rosacea patients has been demonstrated to possess distinct characteristics. Quantitatively, it was observed that individuals with rosacea were 13-fold more likely to develop small intestinal bacterial overgrowth (SIBO) compared to the control group (23). SIBO has been proposed to augment intestinal permeability (24). This, in turn, may lead to the translocation of bacterial components and proinflammatory cytokines into the systemic circulation, subsequently triggering skin inflammation (24, 25). The presence of SIBO might provoke rosacea by amplifying levels of TNF or other cytokines, inhibiting IL-17, and initiating the T helper 1-mediated immune response (26, 27). When evaluating the gut microbiome diversity between rosacea subjects and healthy controls, varying results have been observed across studies. One study reported a pronounced decrease in fecal microbial α-diversity when utilizing the Chao 1 index and observed OTUs for statistical analysis (28). Conversely, another study found no significant distinction (9). Despite these differences in α-diversity findings, both studies are in agreement regarding the inter-sample diversity of the gut microbiome. They consistently highlight a statistically significant difference in β-diversity between rosacea patients and control groups (9, 28). Yet, the exact mechanism by which the gut microbiota influences rosacea is not fully understood (29). A major theory suggests that gut microbiota play a pivotal role in metabolizing undigestible complex polysaccharides, subsequently producing specific vitamins, notably K and B12, and short-chain fatty acids (SCFAs), with a particular emphasis on butyrate and propionate, and SCFAs not only diminishes the permeability of the intestinal barrier but modulate skin barrier integrity by promoting keratinocyte metabolism and differentiation (30, 31). This theory offers a partial elucidation for the outcomes of our study. Bacterial genus, such as *Butyrivibrio, Prevotella_9* and *Ruminococcus gauvreauii*, as significant short-chain fatty acid (SCFA) producers within the gut, might act as protective factors against rosacea, potentially owing to the beneficial effects of SCFAs on skin barrier function (32–34).

To date, the landscape of clinical and foundational studies remains sparse in providing detailed insights into the gut microbiota’s composition in rosacea patients and its consequential impact on the disease. Our MR study illuminated the potential protective roles of *Actinobacteria* and the genus *Butyrivibrio* against rosacea, highlighting their significance despite their relatively minor representation in the commensal bacterial community. Specifically, the *Actinobacteria* phylum is instrumental in maintaining gut homeostasis, notably through its capacity to modulate immune responses (35). This modulation includes the induction of regulatory T-cells, primarily by *Bifidobacteria* species, which play a pivotal role in managing immune-inflammatory and autoimmune reactions. In their investigation into the effects of *Bifidobacterium infantis*—a key *Actinobacteria* member—on the modulation of inflammatory diseases, Groeger et al. demonstrated that a 6–8-week regimen with *Bifidobacterium infantis* substantially reduced plasma C-reactive protein (CRP) levels in patients suffering from ulcerative colitis, psoriasis, and chronic fatigue syndrome (36). Moreover, a statistically significant diminution in TNF-α levels was specifically observed in psoriasis and CFS patients, underscoring the therapeutic potential of targeting gut microbiota in managing inflammatory conditions (36).

However, our conclusions derived from this study using MR analysis markedly differ from the gut microbiota characteristics of rosacea patients as summarized in two prior studies (9, 28). By analyzing fecal samples from 12 Korean patients with rosacea and 251 healthy controls, Nam et al. identified links between rosacea and several changes in the composition of the gut microbiota. This included a reduction in the presence of an unidentified genus within the *Peptococcaceae* family, alongside genera such as Methanobrevibacter, Slackia, Coprobacillus, Citrobacter, and *Desulfovibrio*. On the other hand, an increase was noted in the abundance of the genera *Acidaminococcus* and *Megasphaera*, as well as an unknown genus from the *Lactobacillales* order of an unidentified family (9). Chen et al. conducted a study utilizing high-throughput 16S ribosomal RNA sequencing to compare the fecal microbiomes of 11 rosacea patients with those of 110 non-rosacea subjects (28). The analysis identified a set of enriched genera in rosacea subjects, including *Rhabdochlamydia, CF231, Bifidobacterium, Sarcina, and Ruminococcus, affiliated with the phyla Chlamydiae, Bacteroidetes, Actinobacteria, and Lentisphaerae*. Conversely, a reduction in the abundance of genera such as *Lactobacillus*, *Megasphaerae*, *Acidaminococcus*, *Hemophilus*, *Roseburia*, and *Clostridium*, from the phylum *Firmicutes*, and *Citrobacter*, from *Proteobacteria*, was observed (28). We believe several factors contribute to this discrepancy. Firstly, the causal relationships inferred from MR studies may not equate directly to clinical realities. For instance, an exposure with a strong causal link to the outcome may not be evident in clinical samples if its occurrence is rare. Secondly, the data for our study originates from European populations in the FinnGene, which contrasts with the Asian samples in the two previous cross-sectional studies. Lastly, rosacea treatments, such as antibiotics, have been demonstrated to impact the gut microbiota of patients, which represents a confounding factor that the two prior clinical studies might have struggled to control for (37–39). Consequently, our study delves into the relationship between gut microbiota and rosacea from a different perspective, offering complementary insights to prior research. Looking forward, ongoing research in this area is expected to unveil novel strategies for preventing and treating rosacea, and comprehensive understanding could ultimately inform the development of customized interventions, such as dietary interventions and probiotic supplements, designed to modulate the gut microbiome composition.

Nevertheless, as with any scientific endeavor, this study has limitations. First, it is essential to mention that none of the results from this study met the strict False Discovery Rate correction threshold. Although the results do not meet the stringent threshold, their nominal significance should not be dismissed lightly. Second, we did not differentiate between genders in our analysis. This oversight may have impacted our results, especially considering that rosacea manifest with higher prevalence in women compared to men. Third, the populations in all the GWAS studies were exclusively of European ancestry, introducing a potential for stratification bias. Consequently, our findings might not be directly applicable to populations of other ethnic backgrounds. Last, In the exposure dataset, the most detailed taxonomic level available was the genus. This limitation restricted our ability to explore relationships at the species level.

## Conclusion

In summary, our MR study, in tandem with historical data, underscores the profound connection between gut microbiota and skin health, especially in the context of rosacea. It stresses the importance of considering gut health interventions in the management of rosacea.

## Data availability statement

The original contributions presented in the study are included in the article/Supplementary material, further inquiries can be directed to the corresponding author.

## Author contributions

JL: Conceptualization, Data curation, Methodology, Visualization, Writing – original draft. FY: Conceptualization, Data curation, Validation, Writing – original draft. YL: Visualization, Writing – original draft. XJ: Conceptualization, Writing – review & editing.

## Footnotes

^1^ http://www.phenoscanner.medschl.cam.ac.uk/

## References

[1] Tan J Berg M. Rosacea: current state of epidemiology. J Am Acad Dermatol. (2013) 69:S27–35. doi: 10.1016/j.jaad.2013.04.043, PMID: 24229634

[2] van Zuuren EJ. Rosacea. N Engl J Med. (2017) 377:1754–64. doi: 10.1056/NEJMcp1506630 29091565

[3] Holmes AD Spoendlin J Chien AL Baldwin H Chang ALS. Evidence-based update on rosacea comorbidities and their common physiologic pathways. J Am Acad Dermatol. (2018) 78:156–66. doi: 10.1016/j.jaad.2017.07.055, PMID: 29089181

[4] Zaidi AK Spaunhurst K Sprockett D Thomason Y Mann MW Fu P. Characterization of the facial microbiome in twins discordant for rosacea. Exp Dermatol. (2018) 27:295–8. doi: 10.1111/exd.13491, PMID: 29283459 PMC6635131

[5] Rainer BM Thompson KG Antonescu C Florea L Mongodin EF Bui J. Characterization and analysis of the skin microbiota in Rosacea: a case-control study. Am J Clin Dermatol. (2020) 21:139–47. doi: 10.1007/s40257-019-00471-5, PMID: 31502207 PMC8062047

[6] Mahmud MR Akter S Tamanna SK Mazumder L Esti IZ Banerjee S. Impact of gut microbiome on skin health: gut-skin axis observed through the lenses of therapeutics and skin diseases. Gut Microbes. (2022) 14:2096995. doi: 10.1080/19490976.2022.2096995, PMID: 35866234 PMC9311318

[7] Lee SY Lee E Park YM Hong SJ. Microbiome in the gut-skin Axis in atopic dermatitis. Allergy, Asthma Immunol Res. (2018) 10:354–62. doi: 10.4168/aair.2018.10.4.354, PMID: 29949831 PMC6021588

[8] Olejniczak-Staruch I Ciążyńska M Sobolewska-Sztychny D Narbutt J Skibińska M Lesiak A. Alterations of the skin and gut microbiome in psoriasis and psoriatic arthritis. Int J Mol Sci. (2021) 22:3998. doi: 10.3390/ijms22083998 33924414 PMC8069836

[9] Nam JH Yun Y Kim HS Kim HN Jung HJ Chang Y. Rosacea and its association with enteral microbiota in Korean females. Exp Dermatol. (2018) 27:37–42. doi: 10.1111/exd.13398, PMID: 28636759

[10] Manzhalii E Hornuss D Stremmel W. Intestinal-borne dermatoses significantly improved by oral application of *Escherichia coli* Nissle 1917. World J Gastroenterol. (2016) 22:5415–21. doi: 10.3748/wjg.v22.i23.5415, PMID: 27340358 PMC4910662

[11] Richmond RC Davey SG. Mendelian randomization: concepts and scope. Cold Spring Harb Perspect Med. (2022) 12:a040501. doi: 10.1101/cshperspect.a040501, PMID: 34426474 PMC8725623

[12] Burgess S Daniel RM Butterworth AS Thompson SG. Network Mendelian randomization: using genetic variants as instrumental variables to investigate mediation in causal pathways. Int J Epidemiol. (2015) 44:484–95. doi: 10.1093/ije/dyu176, PMID: 25150977 PMC4469795

[13] Davey Smith G Holmes MV Davies NM Ebrahim S. Mendel's laws, Mendelian randomization and causal inference in observational data: substantive and nomenclatural issues. Eur J Epidemiol. (2020) 35:99–111. doi: 10.1007/s10654-020-00622-7 32207040 PMC7125255

[14] Kurilshikov A Medina-Gomez C Bacigalupe R Radjabzadeh D Wang J Demirkan A. Large-scale association analyses identify host factors influencing human gut microbiome composition. Nat Genet. (2021) 53:156–65. doi: 10.1038/s41588-020-00763-1, PMID: 33462485 PMC8515199

[15] Burgess S Thompson SG. Avoiding bias from weak instruments in Mendelian randomization studies. Int J Epidemiol. (2011) 40:755–64. doi: 10.1093/ije/dyr036, PMID: 21414999

[16] Burgess S Thompson SG. Interpreting findings from Mendelian randomization using the MR-egger method. Eur J Epidemiol. (2017) 32:377–89. doi: 10.1007/s10654-017-0255-x, PMID: 28527048 PMC5506233

[17] Verbanck M Chen CY Neale B Do R. Detection of widespread horizontal pleiotropy in causal relationships inferred from Mendelian randomization between complex traits and diseases. Nat Genet. (2018) 50:693–8. doi: 10.1038/s41588-018-0099-7, PMID: 29686387 PMC6083837

[18] van Zuuren EJ Arents BWM van der Linden MMD Vermeulen S Fedorowicz Z Tan J. Rosacea: new concepts in classification and treatment. Am J Clin Dermatol. (2021) 22:457–65. doi: 10.1007/s40257-021-00595-7, PMID: 33759078 PMC8200341

[19] Wilkin J Dahl M Detmar M Drake L Feinstein A Odom R. Standard classification of rosacea: report of the National Rosacea Society expert committee on the classification and staging of Rosacea. J Am Acad Dermatol. (2002) 46:584–7. doi: 10.1067/mjd.2002.120625, PMID: 11907512

[20] Tapiainen T Paalanne N Tejesvi MV Koivusaari P Korpela K Pokka T. Maternal influence on the fetal microbiome in a population-based study of the first-pass meconium. Pediatr Res. (2018) 84:371–9. doi: 10.1038/pr.2018.29, PMID: 29538354

[21] Zmora N Suez J Elinav E. You are what you eat: diet, health and the gut microbiota. Nat Rev Gastroenterol Hepatol. (2019) 16:35–56. doi: 10.1038/s41575-018-0061-2, PMID: 30262901

[22] Lee YB Byun EJ Kim HS. Potential role of the microbiome in acne: a comprehensive review. J Clin Med. (2019) 8. doi: 10.3390/jcm8070987 PMC6678709 31284694

[23] Parodi A Paolino S Greco A Drago F Mansi C Rebora A. Small intestinal bacterial overgrowth in rosacea: clinical effectiveness of its eradication. Clin Gastroenterol Hepatol: Official Clin Prac J American Gastroenterolog Assoc. (2008) 6:759–64. doi: 10.1016/j.cgh.2008.02.054 18456568

[24] Takakura W Pimentel M. Small intestinal bacterial overgrowth and irritable bowel syndrome - an update. Front Psychol. (2020) 11:664. doi: 10.3389/fpsyt.2020.00664, PMID: 32754068 PMC7366247

[25] Wang FY Chi CC. Association of rosacea with inflammatory bowel disease: a MOOSE-compliant meta-analysis. Medicine. (2019) 98:e16448. doi: 10.1097/MD.0000000000016448, PMID: 31593075 PMC6799824

[26] Weinstock LB Steinhoff M. Rosacea and small intestinal bacterial overgrowth: prevalence and response to rifaximin. J Am Acad Dermatol. (2013) 68:875–6. doi: 10.1016/j.jaad.2012.11.038, PMID: 23602178

[27] Hayran Y Şen O Fırat Oğuz E Yücel Ç Eren F Külcü Çakmak S. Serum IL-17 levels in patients with rosacea. J Cosmet Dermatol. (2022) 21:1147–53. doi: 10.1111/jocd.14169 33877738

[28] Chen YJ Lee WH Ho HJ Tseng CH Wu CY. An altered fecal microbial profiling in rosacea patients compared to matched controls. Journal of the Formosan medical association =. Taiwan yi zhi. (2021) 120:256–64. doi: 10.1016/j.jfma.2020.04.034, PMID: 32446756

[29] Kim HS. Microbiota in Rosacea. Am J Clin Dermatol. (2020) 21:25–35. doi: 10.1007/s40257-020-00546-8, PMID: 32914214 PMC7584533

[30] Trompette A Pernot J Perdijk O Alqahtani RAA Domingo JS Camacho-Muñoz D. Gut-derived short-chain fatty acids modulate skin barrier integrity by promoting keratinocyte metabolism and differentiation. Mucosal Immunol. (2022) 15:908–26. doi: 10.1038/s41385-022-00524-9, PMID: 35672452 PMC9385498

[31] LeBlanc JG Milani C de Giori GS Sesma F van Sinderen D Ventura M. Bacteria as vitamin suppliers to their host: a gut microbiota perspective. Curr Opin Biotechnol. (2013) 24:160–8. doi: 10.1016/j.copbio.2012.08.005, PMID: 22940212

[32] Bai Y Zhou X Li N Zhao J Ye H Zhang S. In vitro fermentation characteristics and Fiber-degrading enzyme kinetics of cellulose, Arabinoxylan, β-glucan and Glucomannan by pig fecal microbiota. Microorganisms. (2021) 9:1071. doi: 10.3390/microorganisms9051071, PMID: 34065679 PMC8156825

[33] Molinero N Conti E Walker AW Margolles A Duncan SH Delgado S. Survival strategies and metabolic interactions between Ruminococcus gauvreauii and Ruminococcoides bili, isolated from human bile. Microbiology spectrum. (2022) 10:e0277621. doi: 10.1128/spectrum.02776-21, PMID: 35863028 PMC9431564

[34] Miller TL Jenesel SE. Enzymology of butyrate formation by *Butyrivibrio fibrisolvens*. J Bacteriol. (1979) 138:99–104. doi: 10.1128/jb.138.1.99-104.1979, PMID: 35524 PMC218243

[35] Lyons A O'Mahony D O'Brien F MacSharry J Sheil B Ceddia M. Bacterial strain-specific induction of Foxp3+ T regulatory cells is protective in murine allergy models. Clin Experiment Allergy: J British Society for Allergy and Clin Immunol. (2010) 40:811–9. doi: 10.1111/j.1365-2222.2009.03437.x, PMID: 20067483

[36] Groeger D O'Mahony L Murphy EF Bourke JF Dinan TG Kiely B. Bifidobacterium infantis 35624 modulates host inflammatory processes beyond the gut. Gut Microbes. (2013) 4:325–39. doi: 10.4161/gmic.25487, PMID: 23842110 PMC3744517

[37] Angelakis E Million M Kankoe S Lagier JC Armougom F Giorgi R. Abnormal weight gain and gut microbiota modifications are side effects of long-term doxycycline and hydroxychloroquine treatment. Antimicrob Agents Chemother. (2014) 58:3342–7. doi: 10.1128/AAC.02437-14, PMID: 24687497 PMC4068504

[38] Ramirez J Guarner F Bustos Fernandez L Maruy A Sdepanian VL Cohen H. Antibiotics as major disruptors of gut microbiota. Front Cell Infect Microbiol. (2020) 10:572912. doi: 10.3389/fcimb.2020.572912, PMID: 33330122 PMC7732679

[39] Clanner-Engelshofen BM Bernhard D Dargatz S Flaig MJ Gieler U Kinberger M. S2k guideline: Rosacea. J Dtsch Dermatol Ges. (2022) 20:1147–65. doi: 10.1111/ddg.14849, PMID: 35929658

