# Supplementary material for: Causal relationship between gut microbiota and rosacea: a two-sample Mendelian randomization study
Source: Front Med (Lausanne). 2024 Mar 22;11:1322685. doi: 10.3389/fmed.2024.1322685 (PMC10995375; doi:10.3389/fmed.2024.1322685)
Supplement: Supplementary file 4 [file Data_Sheet_1.pdf]

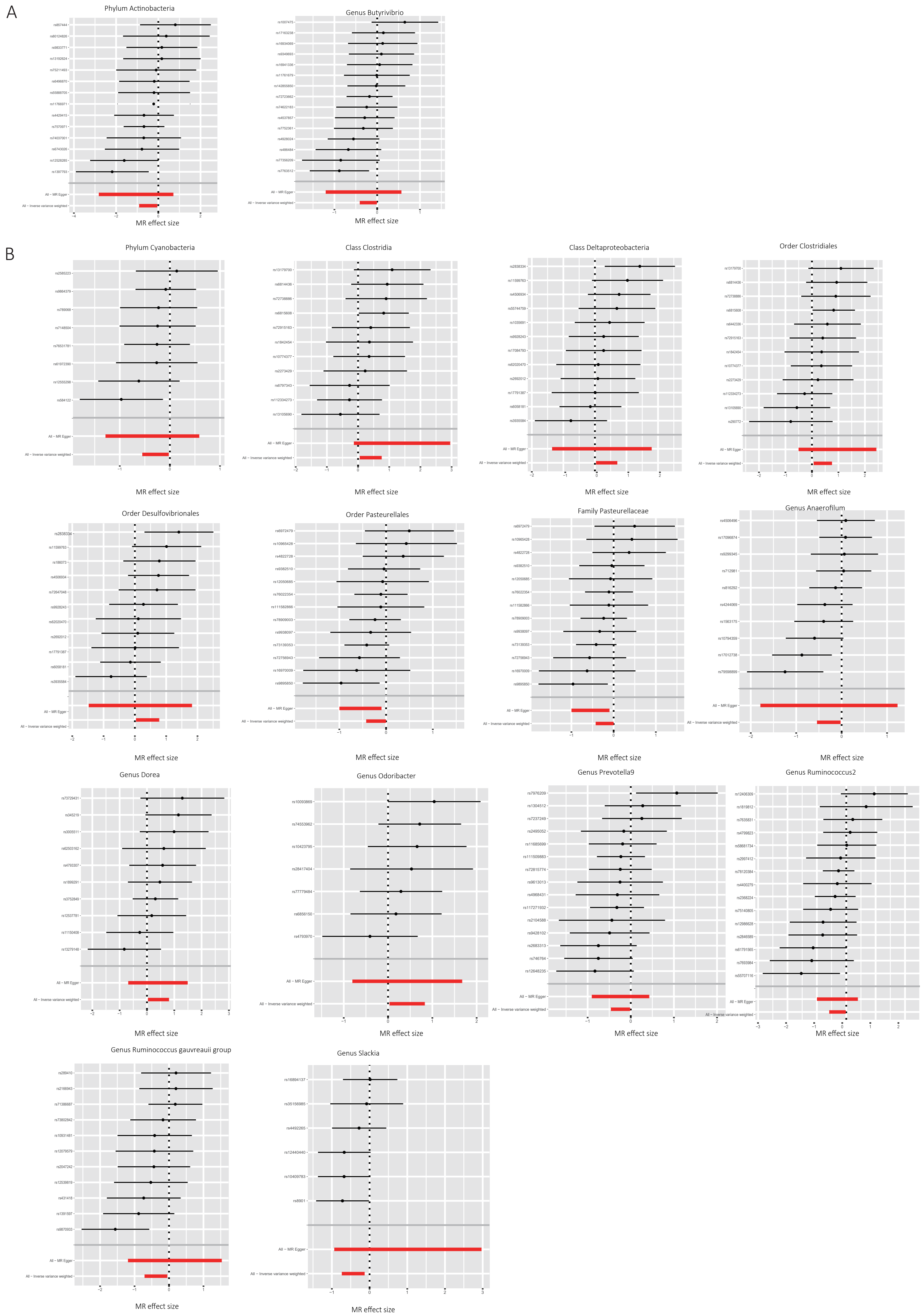

Figure S1. Forest plots for the causal association between several bacteria associated with rosacea (A) and other or unpecified rosacea (B).

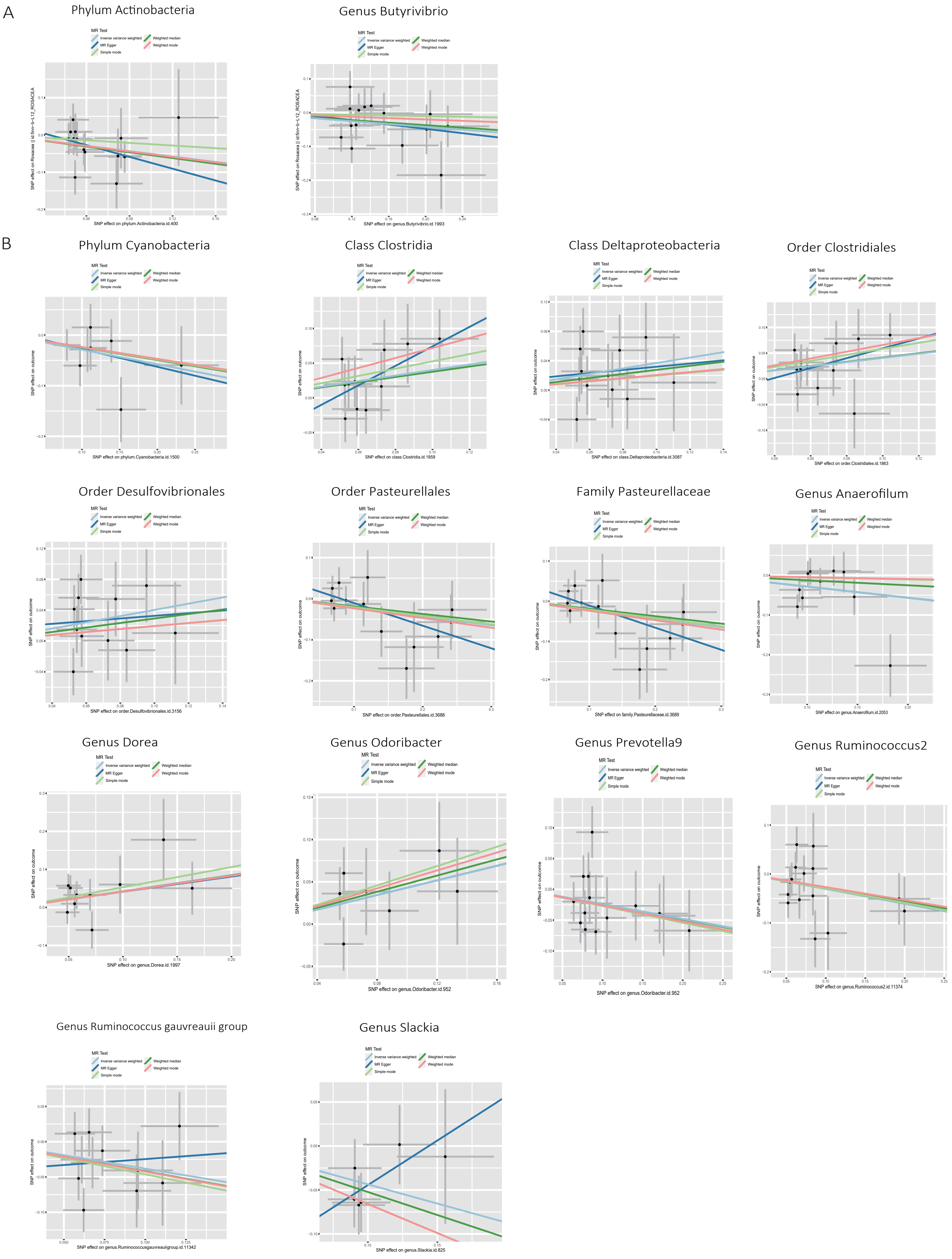

Figure S2. Scatter plots for the causal association between several bacteria associated with rosacea (A) and other or unpecified rosacea (B).

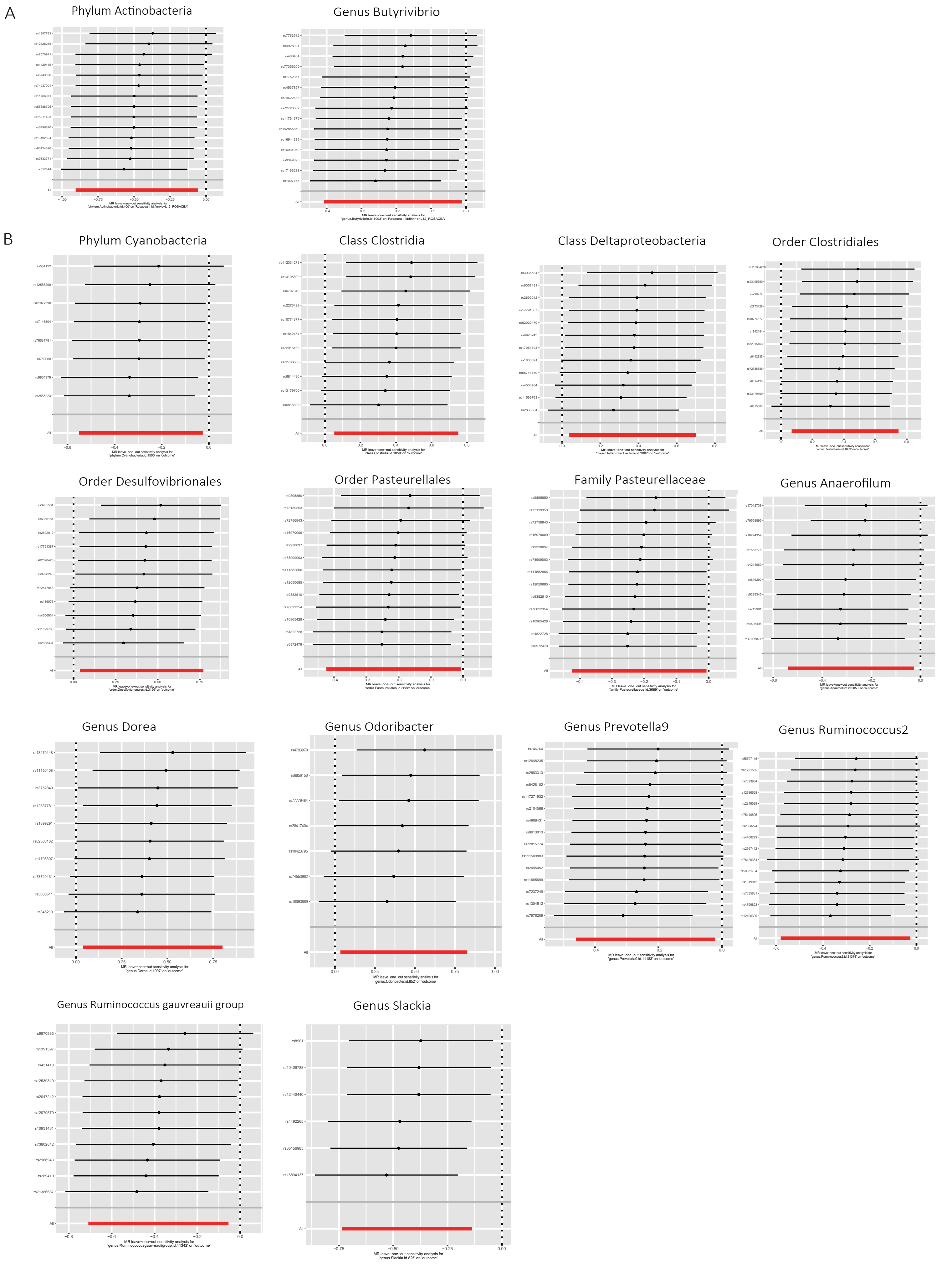

Figure S3. | Leave-one-out analysis of thethe causal association between several bacteria associated with rosacea (A) and other or unpecified rosacea (B).
